# A Transgenic Syrian Hamster Model for New World Hemorrhagic Fever Mammarenavirus Infection

**DOI:** 10.21203/rs.3.rs-9172948/v1

**Published:** 2026-03-31

**Authors:** Samantha R. Wasson, Yanan Liu, Minghao Li, Kevin W. Bailey, Jonna B. Westover, Julio Landinez-Aponte, Arnaud Van Wettere, Venkatraman Siddharthan, Christopher J. Davies, Tracy R. Daniels-Wells, Rong Li, Manuel L. Penichet, Zhongde Wang, Brian B. Gowen

**Affiliations:** Utah State University; Utah State University; Utah State University; Utah State University; Utah State University; Utah State University; Utah State University; Utah State University; Utah State University; University of California, Los Angeles; Utah State University; University of California, Los Angeles; Utah State University; Utah State University

**Keywords:** arenavirus, mammarenavirus, Junín virus, hamster model, transferrin receptor 1

## Abstract

Mammarenaviruses are endemic in rodent populations worldwide, and their zoonotic transmission can lead to a severe, life-threatening hemorrhagic fever (HF) syndrome. In the Americas, five New World mammarenaviruses (NWMs) are known to cause HF. In the absence of FDA-licensed vaccines or antiviral therapies, these viruses pose a significant public health risk and a threat to national security. Research into NWM pathogenesis and the development of effective countermeasures is severely limited by the lack of small-animal models that faithfully reproduce human disease. The pathogenic NWMs use human transferrin receptor 1 (hTfR1) as the receptor for cellular entry. Here, we describe the development of an hTfR1 knock-in (KI) golden Syrian hamster line and its susceptibility to Junín virus (JUNV) infection. This transgenic hamster infection model supports TfR1-mediated entry as an essential step in JUNV pathogenesis and provides a novel small-animal model to advance the development of promising host receptor-directed therapies and direct-acting antivirals to improve outcomes in patients with severe disease caused by pathogenic NWMs.

## INTRODUCTION

Several pathogenic New World mammarenaviruses (NWMs) pose significant threats to public health and national security^1,2^. Mammarenaviruses are endemic in rodent populations worldwide, and their zoonotic transmission can lead to a severe, life-threatening hemorrhagic fever (HF) syndrome, for which no FDA-licensed vaccines or antiviral therapies exist^3–5^. At least five NWMs are known to cause HF in South America, and one is reportedly associated with HF in the US^4,6,7^. Patients infected with Junín virus (JUNV), a prominent pathogenic NWM endemic to Argentina, can present with severe symptoms, including fever, myalgia, hemorrhage (especially near mucus membranes), and neurological manifestations such as confusion, ataxia, and tremors^8–10^. Underscoring the severity of JUNV infections, Argentine HF has a case fatality rate of 15–30%^5,8^.

Research to understand NWM pathogenesis and develop effective countermeasures has been hindered by the lack of small-animal models that are representative of human disease^11,12^. Animal models are essential to gaining insights into viral pathogenesis and evaluating promising therapeutic interventions. Models utilizing macaques or marmosets are considered the “gold standard” for studying JUNV infection as they closely replicate human disease. However, these models are costly and require specialized housing and care within high-level biocontainment facilities that are not available to most researchers. Most standard laboratory rodent species, such as mice and hamsters, are refractory to disease caused by infection with JUNV and other pathogenic NWMs^11^. Mice defective in type I and type II interferon response pathways manifest varying degrees of susceptibility. Still, these models are based on viral challenge of immunocompromised hosts and fail to replicate hemorrhagic disease manifestations^13–15^. The only immunocompetent, commercially available small mammal susceptible to JUNV, Machupo, and Guanarito NWMs is the guinea pig. Specifically, the Hartley guinea pig has served as the primary animal model to investigate NWM pathogenesis and for screening promising therapeutic agents and vaccines^16–27^. The guinea pig models, however, do not reproduce key prominent features of pathogenic NWM hemorrhagic disease in humans, such as hemorrhagic manifestations and neurological symptoms, including ataxia and cerebellar tremors^8,10,28^.

Pathogenic NWMs infect cells of their natural rodent reservoir species via the transferrin receptor 1 (TfR1)^29,30^. These viruses have, in addition, adapted to utilize human TfR1 (hTfR1) as the entry receptor for human cells^31^. hTfR1 is also used by a related North American NWM member of the Whitewater Arroyo virus species complex^32^, which is suspected in fatal HF cases in the United States^7,33^. Thus, the use of hTfR1 appears to be a feature specific to the pathogenic NWMs. Related NWMs that enter through TfR1-independent mechanisms do not cause disease in humans^34^. Moreover, species known to be susceptible to disease when challenged with pathogenic HF-causing NWMs (guinea pigs, marmosets, macaques) express TfR1 orthologs that are capable of mediating attachment and cellular entry^35,36^. By contrast, species that are refractory to pathogenic NWM infection (conventional laboratory mice and hamsters) express TfR1 orthologs that do not support virus entry^29,35^. In summary, these findings indicate that the ability of NWMs to use TfR1 for entry is crucial in defining species vulnerability to severe disease^37^.

Previous work found that homozygous knock out of murine TfR1, while knocking in the human ortholog, renders laboratory mice susceptible to lethal disease following intraperitoneal (i.p.) challenge with the Romero strain of JUNV^38^. Further characterization of mice expressing human (h)TfR1 found that susceptibility to JUNV was age- and dose-dependent, with the mice becoming refractory to severe disease by five to six weeks of age^38^. Although hTfR1 expression confers susceptibility to lethal JUNV disease in young mice, this mouse model does not recapitulate the hemorrhagic manifestations of human disease. And while the hTfR1 mouse model is appropriate for proof-of-concept studies evaluating antiviral drug candidates that target viral entry and replication machinery, a model that is more representative of viral hemorrhagic fever would be better suited for the study of NWM pathogenesis and therapies to mitigate vascular leakiness, considered the fatal lesion in severe cases of disease^39,40^.

Golden Syrian hamsters are a promising small animal model for arenaviral HF. When challenged with Pirital virus (PIRV), a NWM member of the Tacaribe complex that does not use hTfR1 for cellular entry nor is associated with human disease, the infection was lethal, and the hamsters developed coagulopathy and other hemorrhagic manifestations^41,42^. In addition, the closely related Pichindé virus (PICV), which is not pathogenic in humans, also causes lethal HF-like disease in hamsters, including vascular leak syndrome^43^, a cardinal feature of viral HF^44^. Hamsters also recapitulate Marburg HF, showing petechial rash and internal hemorrhage when challenged with Marburg virus, a filovirus related to the Ebola virus^45^. To investigate whether the presence of hTfR1 would render hamsters susceptible to severe JUNV infection with hemorrhagic manifestations, we engineered hamsters expressing hTfR1 under the control of the hamster TfR1 promoter. While these new transgenic animals show susceptibility to JUNV infection, the disease was limited to weight loss and neurologic signs (tremors and ataxia), with low mortality and the absence of hemorrhagic manifestations. The prominent neurological manifestations were consistent with infectious JUNV loads and viral antigen-positive neurons in the brains of moribund animals.

## RESULTS

### Hemorrhagic fever manifestations in hamsters challenged with PICV

**Figure 1:**
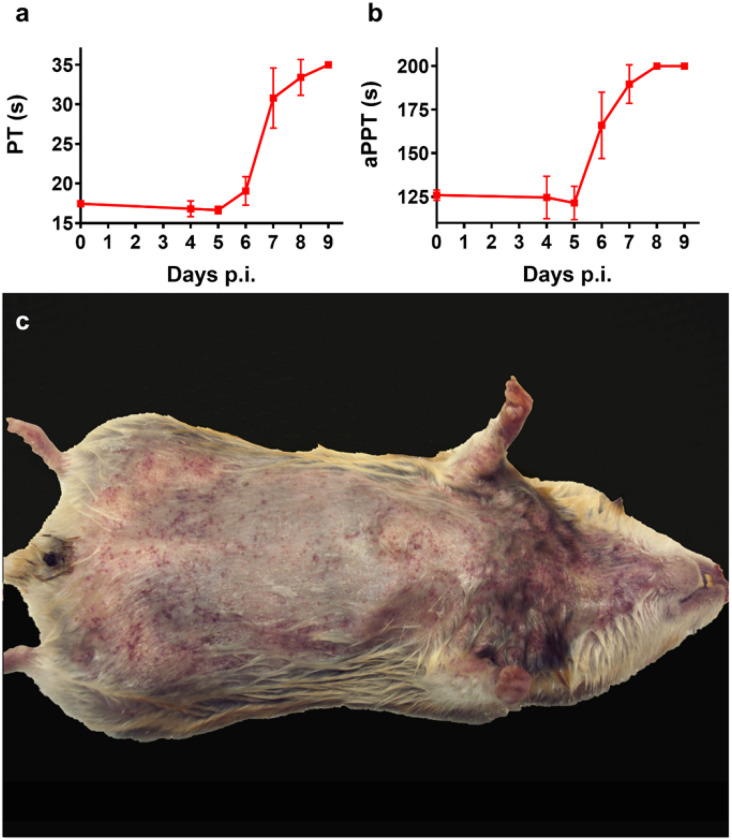
Alteration of coagulation parameters and clinical signs of hemorrhagic fever during PICV infection in hamsters. **a** PT and **b** aPTT were assessed following i.p. infection with 100 CCID_50_ of PICV (*n*=2 for day 0 baseline, *n*=3/group for days 4–7, and *n*=2/group for days 8 and 9 due to one animal from each group succumbing prior to the time of sample collection). Data are reported as group means and standard deviations or spreads. **c** Representative petechia commonly observed on the thorax during advanced infection. Image captured on day 9 p.i. Hamsters generally begin to succumb to hemorrhagic fever-like disease 8–9 days post PICV challenge.

To assess the extent to which hamsters develop coagulopathy and hemorrhaging observed in cases of viral HF caused by NWMs, wild-type (WT) hamsters were challenged i.p. with 100 CCID_50_ (median infectious dose) of PICV, a NWM non-pathogenic to humans but lethal to hamsters^46^. Alterations in prothrombin (PT) and activated partial thromboplastin (aPTT) times were assessed at various time points post-infection (p.i.). Starting on day 6 p.i., PT and aPTT times increased dramatically compared to the normal range values measured on days 0, 4, and 5 after virus challenge (Fig. 1a and 1b), consistent with the onset of hypercytokinemia (day 6) and vascular leak (day 7) observed in this model^43^. PICV-infected hamsters generally began to succumb to HF-like disease 8–9 days post-challenge. Petechia developed on the thoraces, axillae, abdomens, and inguinal areas of critically ill hamsters during advanced infection (Fig. 1c). These rashes resembled petechia observed on the axillary regions and upper chests of human patients infected with pathogenic NWMs^5,10^.

#### Development of a hTfR1 transgenic hamster line

The inability of hamster TfR1 to support entry by pathogenic NWMs likely contributes to the hamster’s resistance to JUNV infection and disease. Indeed, expression of hTfR1 in the CHO-K1 Chinese hamster kidney fibroblast cell line facilitates JUNV attachment and entry, while expression of Syrian hamster and mouse TfR1 orthologs did not^35^. Based on these data and the severe HF-like disease that follows PIRV and PICV infections (^41,42^; Fig. 1), we pursued the development of a new model of JUNV infection in hamsters genetically engineered to express hTfR1.

**Figure 2:**
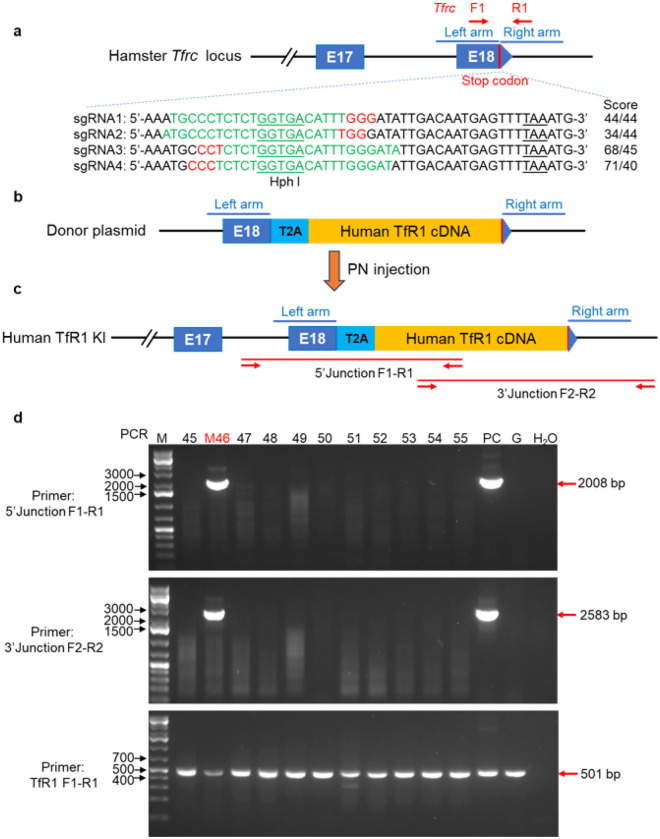
Schematic diagram of the generation of human TfR1 knock-in hamsters through CRISPR/Cas9. **a** sgRNA design targeting hamster *Tfrc* CDS18 locus (Gene ID: 101833501). sgRNA sequence (in green) with PAM highlighted in red, endogenous stop codon underlined in black, on-target/off-target scores on Benchling, and the position of PCR primers (TfR1 F1-R1) and restriction enzyme (HphI, green underlined) used for detecting DNA cleavage efficiency. The stop codon is depicted as a red vertical bar, and the left and right homologous arms are depicted as light blue lines. **b** Construction of donor plasmid pKO2.1-T2A-hTfR1. The hTfR1 cDNA was inserted immediately before the stop codon in exon 18 (E18) of the hamster TfR1 gene. A self-cleaving T2A linker was placed between the stop codon of the hamster *Tfrc* coding region and the hTfR1 insert, enabling expression of both hamster TfR1 and hTfR1 from the endogenous hamster *Tfrc* promoter through ribosomal skipping. **c** Diagram of the genomic locus after successfully inserting the hTfR1 expression cassette into the hamster *Tfrc* locus. Red arrows are genotyping junction primers, 5’ junction F1-R1 and 3’ junction F2-R2, which amplify 2,008 bp and 2,583 bp of the left and right junctions, respectively. **d** PCR genotyping of F0 founder hamsters by junction primers and primer TfR1 F1-R1. The hTfR1 gene insertion was confirmed in the hamster F0M46 line through PCR genotyping. PC, positive control, gDNA from plasmid pKO2.1-T2A-hTfR1 transfection of BHK-21 cells; G: negative control, gDNA from plasmid pmaxGFP transfection; H_2_O negative control.

A CRISPR/Cas9-mediated site-specific homologous recombination technique was used to efficiently knock in the human *TFRC* coding sequence (for hTfR1) into the hamster germline by appending it to the end of the hamster *Tfrc*, as described in Fig. 2a–c. Single guide (sg)RNAs targeting immediately upstream of the hamster *Tfrc* stop codon were constructed for donor cDNA insertion. Among the designed sgRNAs, the sgRNA1 with high cleavage activity and located closest to the integration site was selected for pronuclear (PN) injection with the donor DNA construct (Supplementary Fig. S1a, b). The donor cDNA containing a T2A linker and the hTfR1 coding sequence was delivered to hamster oocytes by pronuclear (PN) injection. Successful insertion of the hTfR1 coding sequence in F0 founders was identified in one animal (male 46; M46) by PCR-based genotyping across the upstream and downstream insertion sites (Fig. 2d). Further genotyping demonstrated that M46 was heterozygous for the hTfR1 coding sequence (Fig. 2d, **lower gel**). The PCR amplification products for hamster M46 were sequenced using the Sanger method^47^. The correct donor sequence was confirmed, except for a Serine-to-Arginine substitution at position 499 (S499R). Notably, the region of hTfR1 that binds to the viral envelope glycoprotein complex (GPC) is far removed from position 499 and, therefore, would not be expected to affect the binding interaction between the pathogenic NWMs and the receptor. With the successful hTfR1 insertion confirmed immediately upstream of the hamster *Tfrc* stop codon, the M46 animal served as the F0 founder for the hTfR1 hamster line. M46 was bred with WT hamsters to produce F1 heterozygous (HET) hTfR1 pups, then F1 animals were crossed to produce homozygous (HOM) hTfR1 pups, which were confirmed through PCR genotyping.

**Figure 3:**
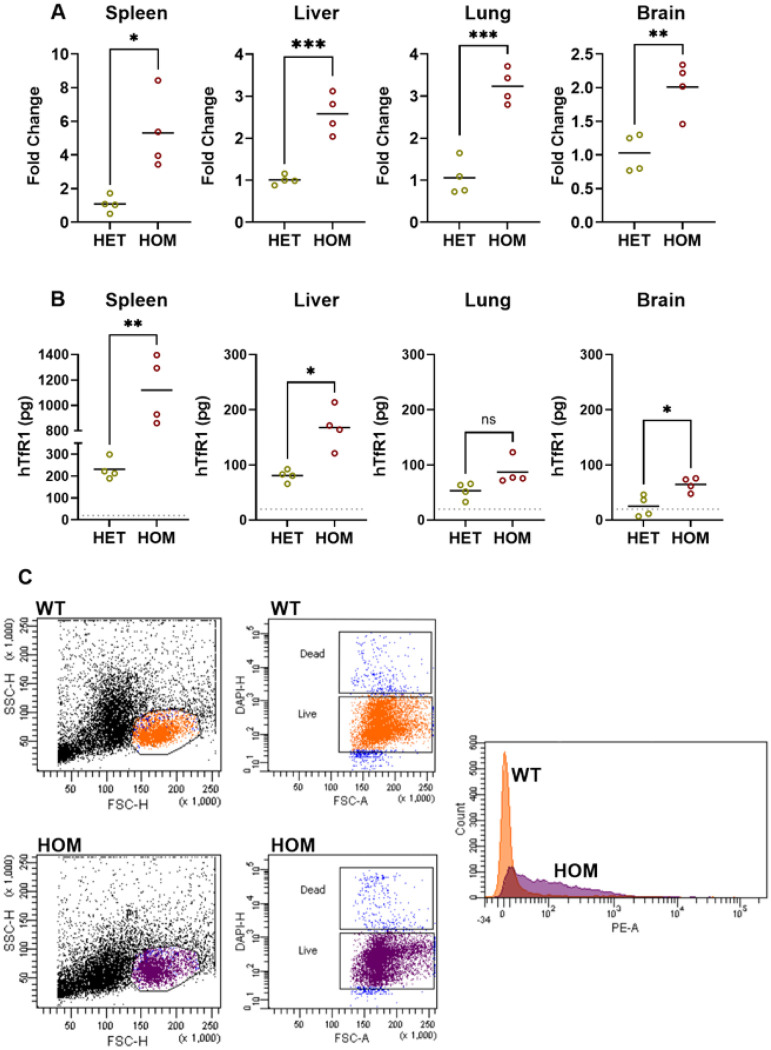
Expression of hTfR1 transgene confirmed in weanling hamsters containing the cDNA insert. **a** Relative mRNA expression (mean) of hTfR1 in selected tissues (spleen, liver, lung, and brain) in HOM or HET hTfR1 hamsters (*n*=4 per group) was measured by RT-qPCR. **b** Production of hTfR1 protein (mean) in tissues from HOM or HET hTfR1 hamsters in selected tissues was compared by quantitative Western blot. **c** Representative cell surface expression of hTfR1 measured by flow cytometry in mixed spleen cell populations of HOM hTfR1 and WT hamsters stained with the ch128.1/IgG1 (mutant Fc-silent) antibody, which binds the apical domain of hTfR1. Forward and side scatter were used to select the larger mononuclear cells, which are predominantly monocytes. Dead cells were excluded from the analysis using Live/Dead staining. *p<0.05, **p<0.01, ***p<0.001, and ****p<0.0001

Expression of hTfR1 mRNA was confirmed and quantified in 3-week-old HET and HOM hTfR1 hamsters using RT-qPCR. HOM hTfR1 hamsters had approximately 2.5-fold more hTfR1 mRNA than HET hTfR1 hamsters in the liver, 5-fold more in the spleen, 3-fold more in the lung, and 2-fold more in the brain (Fig. 3a). To assess hTfR1 expression at the protein level, the tissue samples from the 3 genotypes were examined by Western blotting. Both HET and HOM hTfR1 hamsters had clear hTfR1 expression in spleen, liver, and lung. HOM hTfR1 hamsters produced significantly more hTfR1 protein than HET hTfR1 hamsters in all tested tissues except the lung, where the difference was more subtle. In both hTfR1 genotypes, the spleen produced dramatically more hTfR1 per μg of protein than the other tissues. Brain tissue had the lowest amount of hTfR1 in both HET and HOM hamsters, with 2 of the 4 tested HET samples falling below the limit of detection (Fig. 3b). To verify cell surface expression of hTfR1 in HOM hamsters, mixed spleen cell populations from 3-week-old HOM hTfR1 and WT hamsters were collected, and hTfR1 was detected via immunostaining and flow cytometric analysis. HOM hTfR1 hamsters had a strong signal compared to the background observed in WT hamsters, indicating that hTfR1 proteins were present on the cell surface in HOM hTfR1 hamsters (Fig. 3c). Altogether, these experiments confirmed that hTfR1 was expressed in target tissues and cells of hTfR1 hamsters.

#### Susceptibility of hTfR1 hamsters to JUNV infection

**Figure 4:**
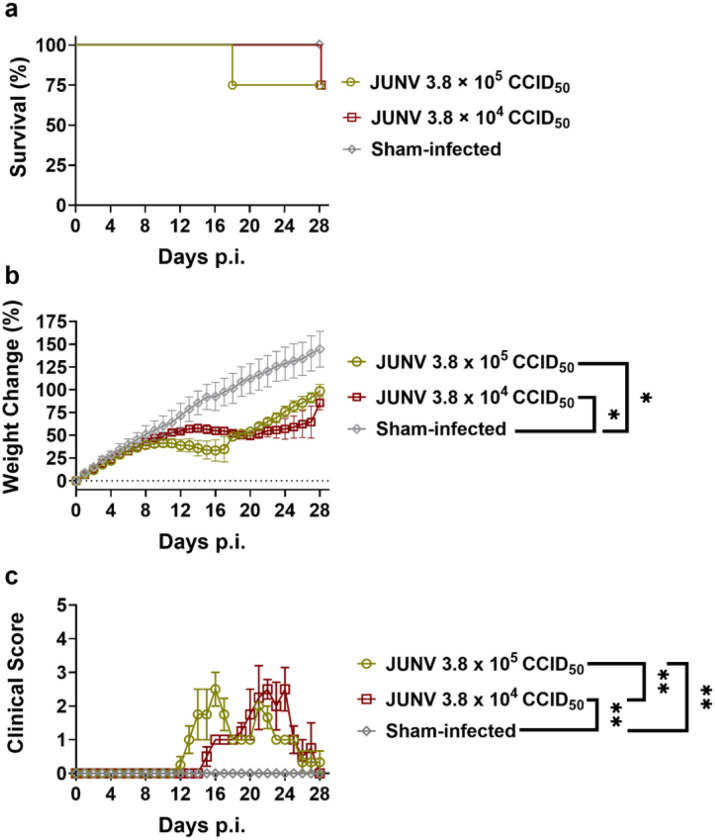
A pilot JUNV challenge study shows that hamsters homozygous for hTfR1 are susceptible to infection and disease. Cohorts of weanling HOM hTfR1 hamsters were infected i.p. with 3.8 × 10^5^ CCID_50_ or 3.8 × 10^4^ CCID_50_ of JUNV (*n*=4 per group), or sham-inoculated with the vehicle (*n*=3). **a** Mortality, **b** weight change, and **c** clinical scores were recorded daily. Mean and standard error of the mean (SEM) are shown in **b** and **c**. *p<0.05 and **p<0.01.

To determine if hTfR1 expression in hamsters confers susceptibility to lethal disease from JUNV infection, 24- to 25-day-old HOM hTfR1 hamsters were challenged i.p. with 3.8 × 10^4^ or 3.8 × 10^5^ CCID_50_ of the pathogenic JUNV Romero strain or sham-infected. Both doses of JUNV resulted in 25% mortality, with 3 of 4 hamsters surviving in each group of infected HOM hTfR1 hamstI jers (Fig. 4a). The animals that succumbed following exposure to the higher and lower challenge doses of virus expired on day 18 and 28 p.i., respectively. Both JUNV-challenged groups experienced weight loss and had lower body weights than the sham-infected controls (Fig. 4b). Notably, the higher-challenge-dose group stagnated approximately 9 days after the infection and collectively lost weight until day 16 p.i. The surviving hamsters in this high-dose group started their recovery approximately 20 days p.i. (Fig. 4b). Weight stagnation and loss were delayed and more modest in the hamsters infected with the lower challenge dose of JUNV. The animals were also scored daily for clinical disease on a scale of 1–5 based on the following signs: weight loss (5% of peak weight), lethargy, ruffled fur, hunched posture, and neurologic signs (tremors and ataxia). Both high and low challenge dose cohorts of HOM hTfR1 hamsters exhibited clinical signs of disease, with the high-dose group’s clinical scores peaking earlier than the hamsters challenged with the lower dose of JUNV (Fig. 4c). However, there was no evidence of a petechial rash seen with PICV in any of the animals. The surviving hamsters were generally in good condition at the conclusion of the 4-week observation period.

**Figure 5:**
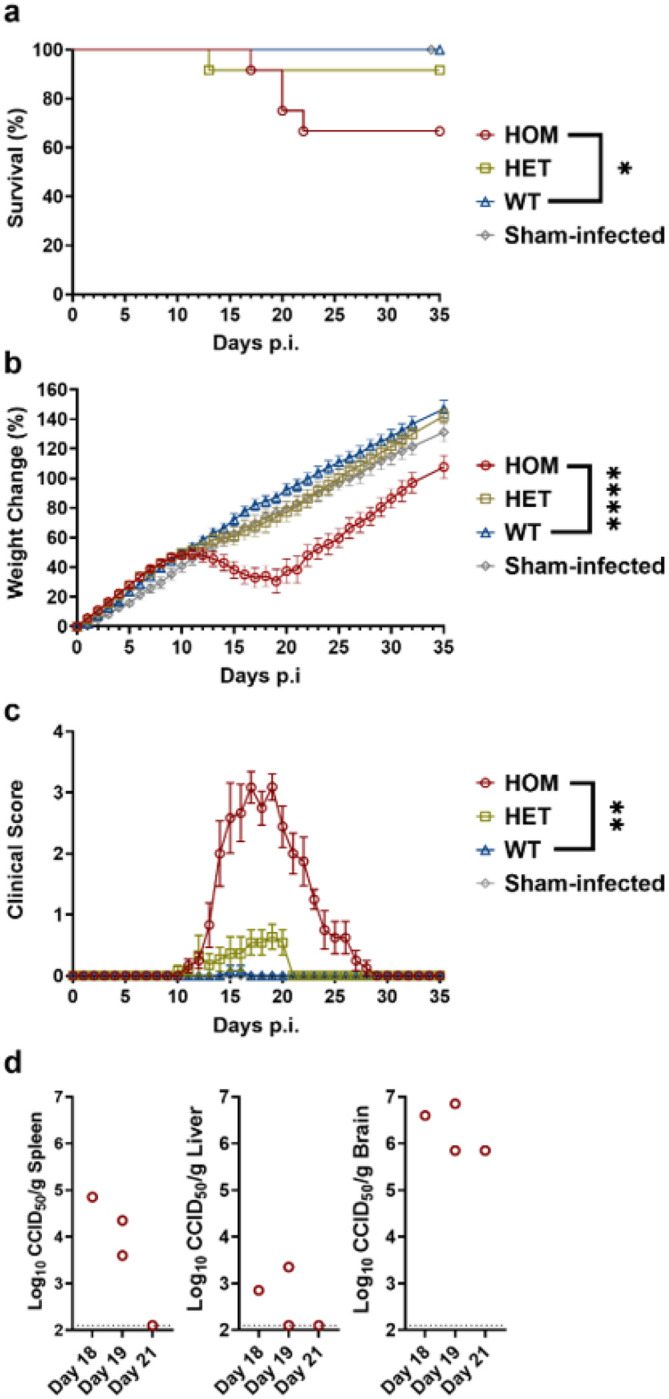
Analysis of survival, weight change, clinical signs, and viral loads in HOM and HET hTfR1 hamsters challenged with JUNV. Weanling HOM hTfR1, HET hTfR1, and WT hamsters (*n*=12 per group) were challenged i.p. with 7.0 × 10^4^ CCID_50_ of JUNV. Sham-infected WT hamsters (*n*=2) were inoculated with an equal volume of the vehicle. **a** Mortality, **b** weight change, and **c** clinical scores were observed for 35 days p.i. **d** Liver, spleen, and brain tissues were collected from several moribund HOM hTfR1 hamsters (*n*=4) and assessed for infectious JUNV viral titers. *p<0.05, **p<0.01, and ****p<0.0001 compared to WT hamsters.

After the initial pilot infection study (Fig. 4), the link between hTfR1 expression and hamster disease was further explored using both HOM and HET hTfR1 hamsters. In the follow-up experiment, cohorts of 24-day-old HOM and HET hTfR1 hamsters and WT control hamsters were inoculated i.p. with 7 × 10^4^ CCID_50_ of JUNV. Consistent with our initial findings, a 33% (4 of 12) mortality rate and uniform, remarkable weight loss were observed in the JUNV-infected HOM hTfR1 hamsters (Fig. 5a, b). In the HET hTfR1 hamster cohort, all except one animal survived, and only mild indications of disease and more rapid recovery were observed (Fig. 5a–c). As expected, WT hamsters were refractory to JUNV infection and disease. In contrast, all JUNV-challenged HOM hTfR1 hamsters became clinically ill (weight loss, lethargy, ruffled fur, and/or hunched posture) and presented with varying degrees of tremors and ataxia, ranging from mild to severe. As in the pilot study, no petechiae were observed.

To investigate the pathogenesis of JUNV infection in the hTfR1 hamsters, liver, spleen, and brain samples were collected from the 4 moribund HOM hTfR1 hamsters to assess viral burden and histopathology in target organs. In agreement with the severe tremors and ataxia observed in the moribund hamsters, the brain had the highest concentration of infectious JUNV titers, approximating 6 to 7 log_10_ CCID_50_/g of tissue, with undetectable to modest titers present in the spleen, and to a lesser extent, the liver (Fig. 5d).

**Figure 6:**
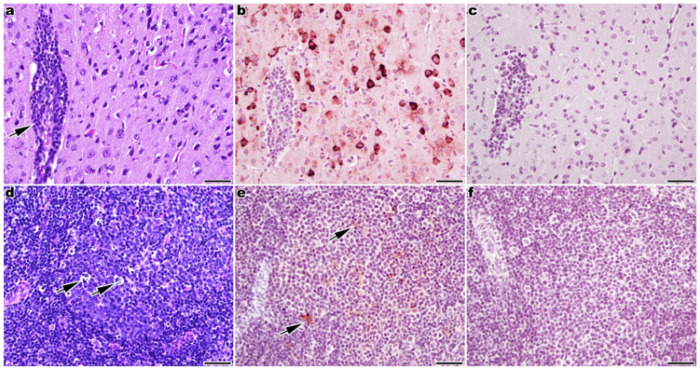
Histopathology and JUNV NP antigen detection in tissues collected from infected, moribund HOM hTfR1 hamsters. Brain and spleen tissue sections from moribund HOM hTfR1 hamsters. **a** Perivascular infiltration of lymphocytes (arrow) in the brain and **b** presence of JUNV NP in many neurons (dark red staining). **d** Individual lymphocyte necrosis (arrows) in the spleen and **e**JUNV NP in mononuclear cells (lymphocytes and/or macrophages) in a splenic follicle germinal center (arrows). **a**, **d** Hematoxylin and eosin staining. **b**,**e** JUNV NP detected by IHC. **c**, **f**No primary antibody IHC controls. **b,c,e,f** Hematoxylin counterstaining. 400× magnification; scale bar = 50 μm.

In brain tissue sections from moribund HOM hTfR1 hamsters, encephalitis with perivascular infiltration of lymphocytes (Fig. 6a) and the presence of JUNV nucleoprotein (NP) within neurons (Fig. 6b) were observed by histology and immunohistochemistry (IHC), respectively. Histologic examination of the spleen showed lymphocyte necrosis in splenic follicle germinal centers (Fig. 6d), and IHC demonstrated JUNV NP within mononuclear cells in germinal centers, which could indicate infection of lymphocytes, macrophages, or both (Fig. 6e). There were no histologic lesions in liver or lung tissue sections (not shown).

## Discussion

Pathogenic NWMs primarily rely on a compatible TfR1 for entry into host cells via cell-mediated endocytosis^31^. Species with TfR1 orthologs known to be compatible with NWMs include humans, several non-human primates (including macaques and marmosets)^48,49^, guinea pigs^35^, and JUNV’s reservoir rodent host, the drylands vesper mouse^50^. Laboratory mice are not susceptible to NWM disease, but when genetically modified to express hTfR1, they become susceptible to JUNV disease when challenged at approximately 3 to 4 weeks of age^38^. However, the age-dependent susceptibility and the lack of HF manifestations limit the utility of this mouse model. Based on the coagulopathy, petechial rash, and vascular leak observed in hamsters infected with PICV (and the related PIRV), we genetically engineered hamsters encoding hTfR1 and assessed their susceptibility to JUNV infection and disease, with the goal of developing a new small-animal model of mammarenaviral HF.

To create an hTfR1 hamster model in which hTfR1 expression closely mimics that of the endogenous hamster TfR1 gene both temporally and spatially, we employed a CRISPR-mediated transgenic technique developed by our group to knock in a T2A-hTfR1 cassette into the hamster *Tfrc* locus. This strategy renders hTfR1 expression fully under the control of the endogenous hamster TfR1 promoter and its associated regulatory elements. The T2A peptide linker enables bicistronic expression of endogenous hamster TfR1 and hTfR1 from the same mRNA, while allowing their translation into independent proteins with equimolar levels^51,52^. As the tissue expression pattern and level of a viral receptor gene are directly relevant to viral infection and pathogenesis, our T2A-mediated approach is designed to ensure that the expression pattern and level of hTfR1 accurately reflect those under normal physiological conditions; this is in contrast to the commonly used plasmid integration approach, in which a transgene randomly integrates into the animal genome and that transgene expression is unpredictable due to genomic position effects^53^. Furthermore, this CRISPR-assisted transgenic strategy avoids the introduction of additional foreign sequences into the hamster TfR1 locus, which would otherwise occur with conventional homologous recombination approaches.

Critical to the performance of the model, we confirmed hTfR1 transgene expression at the mRNA and protein levels, including cell-surface expression in spleen cells, consistent with the expected forward-and side-scatter distributions of monocytes. Based on the age-dependent susceptibility of hTfR1 mice to JUNV, we challenged weanling hTfR1 hamsters in an initial pilot study and observed limited mortality (25%), with all animals exhibiting various degrees of tremor and ataxia. Subsequently, a study with larger cohorts, including HET littermates, confirmed the low mortality (33%) and the uniform presentation of tremors and ataxia in HOM hTfR1 hamsters. Our findings clearly highlight the importance of hTfR1 expression in the pathogenesis of JUNV infection. They are also consistent with high JUNV titers in brain tissues from moribund hamsters, with modest to undetectable viral loads in spleen and liver samples. Further, JUNV antigen was readily detected in neurons and present in mononuclear cells within splenic follicle germinal centers. Argentine HF, the disease caused by JUNV, has distinct neurological symptoms, including acute tremors, ataxia, memory loss, and a sequela involving neurological complications^5,8,10^. It is possible that the cause of death in the JUNV-infected, hTfR1 hamsters is likely due to neurologic disease, considering the prominent tremors and ataxia, and evidence of high viral titers in brain samples collected from moribund animals. No hemorrhagic manifestations (petechiae, bleeding from mucosal areas) were noted at any time during our daily evaluations of the animals. While the lack of HF-like disease observed with Clade A NWMs (PICV and PIRV) was disappointing, the lower mortality (31%) in the hTfR1 hamsters aligns with the case fatality rate in cases of Argentine HF (15–30%)^5,8^. Presumably, PICV and PIRV replicate more efficiently after cellular entry and/or antagonize the hamster IFN response to the infections, resulting in higher systemic viral loads and HF-like disease^41,43^.

Both the mouse and hamster hTfR1 models of JUNV infection can be used to evaluate proof-of-concept for promising antiviral therapies. Objective efficacy readouts in the mouse model include survival against highly lethal JUNV infection, protection against weight loss associated with acute disease, and reductions in viral burden^38,54^. For the less-lethal hamster hTfR1 model, efficacy would be measured by weight loss reduction as the primary readout, with survival as a secondary assessment if larger cohorts can be enrolled to increase statistical power. Additional studies are needed to assess viral replication kinetics in the spleen and brain to determine whether virological parameters can also serve as measures of antiviral activity. Notably, unlike the guinea pig model, both the hTfR1 mouse and hamster models can be used to assess interventions directed at the apical domain of hTfR1^54^, which mediates attachment and entry by all known pathogenic NWMs^31^. As a specific example of this, the ch128.1/IgG1 antibody that competitively inhibits the binding of the MACV GPC to the apical domain of hTfR1 does not bind the orthologous guinea pig TfR1^36^. Other advantages of the mouse and hamster hTfR1 JUNV challenge models are their amenability to larger-scale studies and the need for less chemical or biological material for initial tolerability and proof-of-concept efficacy evaluations. That said, both the mouse and hamster models have their limitations. Most notably, susceptibility of hTfR1 mice to JUNV infection is agedependent, limiting their use for vaccine trials. Further studies are needed to assess the susceptibility of older hTfR1 hamsters to JUNV. Importantly, we have expanded the “toolbox” by adding the hTfR1 hamster model to the existing hTfR1 mouse and Hartley strain guinea pig JUNV infection models. These immunocompetent small-animal models are complementary, providing flexibility and increasing predictive value through studies across different species.

Our previous research showed that deletion of the *STAT2* gene renders hamsters vulnerable to severe fever with thrombocytopenia syndrome virus (*Bandavirus dabieense*), Heartland virus, Rift Valley fever virus, Marburg virus, and Crimean-Congo hemorrhagic fever virus^55–59^. The *STAT2* deletion impairs type I IFN signaling, ablating transcription cascades induced by IFN-α and IFN-β and limiting MHC-1 expression^59^. Crossing HOM hTfR1 with *STAT2*^*−/−*^ hamsters could yield the desired HF disease model exploiting hTfR1-mediated entry, while interfering with the type I IFN response. However, type I IFN is a driver of severe disease in hTfR1 mice^38^, introducing uncertainty about whether the proposed hamster cross would result in HF-like disease following JUNV challenge. Further exploration of genetic crosses that would include the hTfR1 hamster is planned.

## METHODS

### Viruses and cells

Pichindé virus (PICV), strain An 4763, was obtained from David Gangemi (Clemson University). The virus stock was prepared from a clarified hamster liver homogenate following a single passage in hamsters. The molecular clone of the Romero strain of Junín virus (JUNV) was rescued in BHK-21 cells and kindly provided by Slobodan Paessler (University of Texas Medical Branch)^60^. The JUNV stock was prepared from a single passage in Vero African green monkey kidney cells (CCL-81). Vero cells were purchased from the American Type Culture Collection (ATCC) and maintained in Minimum Essential Medium (MEM) supplemented with 5% fetal bovine serum (Thermo Fisher Hyclone). BHK-21 (CCL-10) golden hamster kidney cells were obtained from ATCC and maintained in Dulbecco’s Modified Eagle Medium (DMEM) supplemented with 10% fetal bovine serum (FBS) (Thermo Fisher Hyclone).

#### Biosafety and ethics

Work with PICV was conducted in AAALAC-accredited animal (A)BSL-2 laboratories. All PICV-related animal procedures complied with USDA guidelines and were conducted under protocol #14550, approved by the Utah State University Institutional Animal Care and Use Committee. Work with pathogenic JUNV was conducted in AAALAC-accredited and Select Agent-approved animal (A)BSL-3 + laboratories and complied with USDA guidelines and were conducted under protocol #12755, approved by the Utah State University Institutional Animal Care and Use Committee. All JUNV animal studies were performed by Candid#1-immunized staff using appropriate powered air-purifying respirators and personal protective equipment.

#### Prothrombin (PT) and activated partial thromboplastin time (aPTT) assessment in PICV-challenged hamsters

Female golden Syrian hamsters (5- to 7-week-old) were obtained from Charles River Laboratories and acclimated for two weeks before use. Hamsters were fed Harlan lab block and given tap water *ad libitum*. Groups (*n* = 3) of hamsters were infected with 100 CCID_50_ of PICV, given bilaterally in two i.p. injections of 0.1 mL, and sequentially sacrificed starting on day 4 p.i. and continuing through day 9. Control animals (*n* = 2) were included to establish baseline values for PT and aPPT clotting times. Blood collection was performed by cardiac puncture and immediately transferred to sodium citrate tubes. The samples were analyzed using PT/aPPT Combination Test Cartridges on a VetScan VSpro Coagulation Analyzer (Zoetis).

### Design and validation of single guide (sg)RNAs for targeted hTfR1 knock-in

Our target insertion site for hTfR1 cDNA in golden Syrian hamsters was immediately upstream of the hamster *Tfrc* stop codon. Through insertion in this region, human TfR1 and hamster TfR1 would be expressed through the same hamster TfR1 promoter. This precise insertion was achieved via a double-strand break induced by sgRNA/Cas9 ribonucleoprotein complexes (RNPs). sgRNAs were designed using a Benchling web tool (https://www.benchling.com/). To identify cleavage efficiency at the intended integration site, four sgRNAs were designed and tested for compatibility with the 3’ end of the hamster *Tfrc* sequence (Fig. 2a). The indel-inducing activities of the sgRNAs were tested by transfecting sgRNA/Cas9 RNPs into BHK-21 cells (golden Syrian hamster-derived kidney fibroblast cell line). The sgRNA activity was measured by PCR-restriction fragment length polymorphism (PCR-RFLP) assays and Sanger sequencing^47^ on genomic DNA (gDNA) isolated from BHK-21 cells 48 h post-RNP transfection. PCR was performed with Ex Taq (Takara) and the following parameters: initial denaturation at 94°C for 30 s followed by 32 cycles of 10 s denaturation at 98°C, 30 s annealing at 58°C, and 30 s extension at 72°C, with a final extension step of 72°C for 10 min. Primers for amplifying the region targeted by the sgRNAs were: TfR1 F1: 5’-AGAATGAGTCACAAGTTAGAGCA-3’, and R1: 5’-ATCTGAGGCGATATCATGAACAC-3’. The restriction enzyme HphI (New England Biolabs) was used for RFLP. PCR products were also sent for Sanger sequencing, and the chromatograms were analyzed by EditCo to determine the cleavage efficiency.

### Construction of hTfR1 donor vector pKO2.1-T2A-hTfR1 and in vitro knock-in evaluation

The DNA donor vector pKO2.1-T2A-hTfR1 was constructed by NEBuilder HiFi DNA assembly (New England Biolabs). The left homologous arm (1 kb of hamster genomic TfR1 sequence immediately upstream (5’) of the stop codon in exon 18 of the hamster *Tfrc*), a T2A peptide sequence linked to the hTfR1 cDNA (amplified from A549 cells), and the right homologous arm (1 kb of hamster genomic sequence immediately downstream (3’) of the KI site) were PCR amplified with a 25 bp overlap on each side and assembled with linearized pKO2.1 vector (Fig. 2b). To evaluate gene KI of the hTfR1 donor construct, 3 μg donor vector pKO2.1-T2A-hTfR1 was co-transfected with sgRNA1/Cas9 RNP (3 μg) into BHK-21 cells. Genomic PCR with primers designed to amplify the left (5’ junction primer F1: 5’-CCGCAGTTTGAAGCTACCAT-3’; R1: 5’-TGTCCCCAGATGAGCATGTC-3’) and right (3’ junction primer F2: 5’-ACTGGTAAACTGGTCCATGC-3’; R2: 5’-CAAGTGCTGACCGTGAGTG-3’) junctions generated by the successful KI event was used to genotype the transfected BHK-21 cells. PCR was performed with LA Taq (Takara) and the following parameters: initial denaturation at 94°C for 1 min, followed by 32 cycles of 10 s denaturation at 98°C and 3 min extension at 68°C, with a final extension step at 72°C for 10 min. After limiting dilution, we identified single-cell-derived BHK-21 cell lines carrying the hTfR1 cDNA (**Suppl. Fig. S1c**). As the cellular expression of hTfR1 amplified JUNV infection, we were confident to move forward with the transgenic hTfR1 hamster^35^.

### Generation of T2A-hTfR1 Syrian hamsters by pronuclear injection

Syrian hamsters were originally obtained from Charles River and bred in-house. Embryo manipulation and pronuclear injection of T2A-hTfR1DNA donor vector were performed as described previously^61,62^. Briefly, to produce hTfR1 knock-in hamsters, donor female hamsters were superovulated by PMSG injection intraperitoneally on the morning of Day 1 of the estrus cycle and then mated with males four days after late afternoon. 18 hours post-mating, the females were euthanized by CO_2_, and zygotes were recovered by oviductal flushing and cultured in HECM-9 (Hamster Embryo Culture Medium-9^63^) at 37.5°C under 10% CO_2_, 5% O_2_, and 85% N_2_. sgRNA1/Cas9 RNP with donor plasmid pKO2.1-T2A-TfR1 (50 ng/μL: 5 ng/μL) was injected into the pronuclei of zygotes, followed by transferring the injected embryos to the oviducts of pseudopregnant recipient hamsters.

To genotype founder hTfR1 hamsters produced from pronuclear injection experiments and animals from descendant generations, genomic DNA was isolated from toe clippings using Qiagen Blood and Tissue Kit (Qiagen). Primers for amplifying the T2A-TfR1 transgenic cassette were the same as those used for in vitro knock-in evaluation, 5’ junction primer F1-R1 and 3’ junction primer F2-R2. Primers to distinguish homozygous and heterozygous knock-in pups were the same as those used to assess editing efficiency: TfR1 F1-R1.

### Assessment of hTfr1 mRNA and protein expression in hTfR1 KI hamsters

Hamster tissues (liver, spleen, lung, and brain) were collected from 2 male and 2 female 3-week-old HOM hTfR1 and HET hTfR1 hamsters, along with one male WT hamster. For mRNA expression analysis, portions of the tissues were homogenized in Buffer RLT Plus from the RNeasy Plus Mini Kit (Qiagen). Total RNA was extracted following Qiagen’s recommendations. Primers and probe targeting hTfR1 were used to detect mRNA expressed from the transgene. The forward primer was 5’- GAA AAT TCA TAT GTC CCT CGT − 3’ and the reverse primer was 5’- ATC AAC TAT GAT CAC CGA GT −3’. The probe sequence was 5’- /56-FAM/ATC ACG CCA GAC TTT GCT /3BHQ_1/ −3’. A primers and probe set for β−2 microglobulin (B2M) was used as an internal control to normalize and minimize variance in RT-qPCR amplification. The forward primer and reverse primer for B2M were 5’- GGC TCA CAG GGA GTT TGT AC −3’ and 5’- TGG GCT CCT TCA GAG TTA TG −3’, respectively. The probe sequence for B2M was 5’- /5Cy5/ CTG CGA CTG /TAO/ATA AAT ACG CCT GCA /3IAbRQSp/ −3’. All RT-qPCR reactions were performed using a QuantStudio 5 Real-Time PCR System (Applied Biosystems) and SensiFAST^™^ Probe No-ROX One-Step Kit (Meridian Bioscience), with the following amplification cycle conditions: reverse transcription at 45°C for 10 min, polymerase activation at 95°C for 2 min, followed by 40 cycles of denaturation at 95°C for 5 sec and annealing/extension at 60°C for 20 sec. Data are expressed as fold change using the method described by Pfaffl^64^.

To assess hTfR1 protein production by Western blot, portions of spleen, liver, lung, and brain were homogenized in cell extraction buffer (Invitrogen) containing Pierce protease inhibitor cocktail, and total protein was quantified using the Pierce BCA Protein Assay (Thermo Fisher Scientific). Samples were combined with Laemmli buffer, boiled for 5 min, and 12 μg of protein per sample was run on PROTEAN TGX Stain-Free Protein Gels in 25 mM Tris, 192 mM glycine, 0.1% SDS, pH 8.3 buffer (Bio-Rad). Gels were exposed to UV light to promote the binding of trihalo-tryptophan to the sample protein. Then, protein was transferred to a nitrocellulose membrane using Trans-Blot Turbo Mini 0.2 μm Transfer Packs (Bio-Rad), and total protein was imaged using a ChemiDoc Imaging System (Bio-Rad). Nitrocellulose membranes were stained with primary antibody ch128.1/IgG1, which targets the apical domain of hTfR1^65^, and goat anti-human IgG Fc horseradish peroxidase-conjugate secondary antibody (Novex), and signal was produced using the Clarity Max Western ECL Substrate Kit (Bio-Rad). Chemiluminescence signal from hTfR1 staining was normalized to total protein, and relative target protein densitometry was calculated using Image Lab software (Bio-Rad). 12.5 ng of soluble TfR1 (Sino Biological) was included as a positive control in each Western blot. Notably, soluble TfR1 has a molecular weight (m.w.) of 77.4 kDa, which is lower than the approximately 90 kDa m.w. of membrane-bound TfR1 monomers, accounting for the difference between positive control TfR1 and hTfR1 expressed from the transgene. The known amount of the positive control soluble TfR1 was used to estimate the amount of hTfR1 in the tested samples, reported as pg per 12 μg of total protein. WT hamster spleen, liver, lung, and brain were blotted alongside HET and HOM hTfR1 hamster as negative controls for background, non-hTfR1 staining. The highest background staining for each WT hamster tissue was defined as the limit of detection for hTfR1 in our Western blot system (Supplementary Fig. S2).

### Assessment of hTfR1 cell surface expression in KI hamsters by flow cytometry

Spleens collected from male and female 3-week-old HOM hTfR1 hamsters and WT littermates (*n* = 2/group) were homogenized to produce spleen cell suspensions. The cell suspensions were stained using the ch128.1/IgG1 Fc-silent mutant antibody modified to inactivate Fc-mediated effector functions^54^ and phycoerythrin (PE)-conjugated Goat F(ab’)2 Anti-Human Kappa-PE secondary antibody (SouthernBiotech). Forward and side scatter were used to select the larger mononuclear cells, which are predominantly monocytes. The LIVE/DEAD Fixable Blue Dead Cell Stain (Thermo Fisher Scientific), which is detected in the DAPI channel, was used to exclude dead cells from the analysis. The stained spleen cell populations were analyzed by flow cytometry using a FACSAria II Cell Sorter (BD Biosciences).

### JUNV hTfR1 hamster challenge experiments

Male and female 24- to 25-day-old HOM hTfR1 hamsters derived from the M46 founder were obtained through breeding, and the KI was confirmed by PCR genotyping as described above. Here and in the follow-up experiment, the animals were fed Harlan Lab Block and tap water *ad libitum*. For the initial challenge study, cohorts of hamsters (*n* = 4/group) were inoculated i.p. (0.2 mL) with 3.8 × 10^4^ or 3.8 × 10^5^ CCID_50_ of JUNV (Romero strain). Three sham-infected controls were inoculated with the MEM vehicle. Following the virus challenge, the hamsters were weighed and observed for morbidity and mortality for 28 days. Each hamster was assigned a daily clinical score from 1–5 based on the number of clinical signs presented: weight loss (5% of peak weight), lethargy, ruffled fur, hunched posture, and neurologic signs (tremors, ataxia). Animals were humanely euthanized if their body weight fell below 70% of their maximum body weight.

In the follow-up challenge experiment, 24-day-old male and female HOM, HET, and WT hamsters (*n* = 12/group) were challenged i.p. with 7.0 × 10^4^ CCID_50_ JUNV. Two sham-infected controls inoculated with the MEM vehicle were included for comparison. All animals were weighed, observed, and assigned a clinical score daily for 35 days following virus challenge. Moribund HOM hTfR1 hamsters were euthanized to assess viral loads and histopathology in selected tissues.

Necropsies were performed on 4 moribund HOM hTfR1 hamsters euthanized 16–21 days post-JUNV challenge. Spleen, liver, lung, and brain samples were collected to assess viral loads, histopathology, and viral antigen distribution by immunohistochemistry (IHC). To measure JUNV burden in the selected tissues, clarified homogenates were assayed for infectious viral titers by endpoint titration in Vero cell culture^66,67^. Briefly, a specific volume of tissue homogenate was serially diluted and added to triplicate wells of subconfluent Vero cell monolayers in 96-well microplates. The plates were observed 8 days p.i., and the median infectious dose (CCID_50_) per gram of tissue was calculated as previously described^68^.

Samples of the spleen, liver, lung, and brain were fixed in 10% neutral-buffered formalin for histological and IHC analyses. Formalin-fixed tissue samples were processed and embedded in paraffin according to routine histologic techniques. Sections, 5-μm thick, were stained with hematoxylin and eosin (H&E) according to standard methods. IHC staining for JUNV nucleoprotein (NP) was performed 24 h after collection. Samples were deparaffinized by passage through xylene, rehydrated in decreasing grades of alcohol (100%, 95%, 75%, 70%, 50%, and 30%) for 5 min each, and washed with water. For heat-induced epitope retrieval, samples were placed in a high-pH antigen retrieval solution (Invitrogen) and heated to 125°C in a decloaking chamber for 4 min. After returning to room temperature (RT), samples were washed with PBS and permeabilized with 0.5% Triton X-100 for 5 min. Samples were blocked with 10% normal goat serum containing 0.2% Triton X-100 in PBS for 60 min at RT, then diluted goat anti-mouse Fab (Jackson ImmunoResearch) for an additional 60 min. After blocking, samples were incubated with the primary antibody, ch128.1/IgG, for 1 h at RT. Samples were then washed in PBS and incubated with an HRP-conjugated goat anti-mouse secondary antibody (Thermo Fisher Scientific) for 45 min at RT. The target signal was developed using the ImmPACT NovaRed peroxidase substrate kit (Vector Labs). After development, samples were washed with PBS and counterstained with Vector Hematoxylin QS (Vector Labs) for 45 sec. Samples were examined by a board-certified veterinary pathologist using light microscopy.

### Statistical analysis

All statistical analyses were conducted using Prism 10 (GraphPad Software). The unpaired two-tailed *t*-test was used to assess differences in mRNA level, Western blot protein densitometry, and viral titer data. Survival data were plotted using the Kaplan-Meier method and analyzed using the log-rank (Mantel-Cox) test. Weight change comparisons were performed by mixed-effects analysis. Clinical scores were compared using Friedman’s test with Dunn’s multiple comparisons test. The sham-infected group, included to establish baseline values in the second JUNV hamster challenge study (Fig. 5), was not included in the groupwise analysis. Results were considered significant if *p* ≤ 0.05.

## Supplementary Material

Supplementary Files

This is a list of supplementary files associated with this preprint. Click to download.

- hTfR1HamsterSupplementary.docx

## Data Availability

Data generated during this study are included in this published article and its supplementary information files.
